# Developing defined substrates for stem cell culture and differentiation

**DOI:** 10.1098/rstb.2017.0230

**Published:** 2018-05-21

**Authors:** Louise Hagbard, Katherine Cameron, Paul August, Christopher Penton, Malin Parmar, David C. Hay, Therése Kallur

**Affiliations:** 1 BioLamina, Löfströms Allé 5A, 172 66 Sundbyberg, Sweden; 2 Medical Research Council Centre for Regenerative Medicine, University of Edinburgh, 5 Little France Drive, Edinburgh EH16 4UU, UK; 3 Icagen, Discovery Biology, Tucson Innovation Center, Oro Valley, AZ 85755, USA; 4 Wallenberg Neuroscience Center, Department of Experimental Medical Science, Lund University, 221 84 Lund, Sweden

**Keywords:** defined substrates, human recombinant laminins, human pluripotent stem cells, cell culture, cell therapy

## Abstract

Over the past few decades, a variety of different reagents for stem cell maintenance and differentiation have been commercialized. These reagents share a common goal in facilitating the manufacture of products suitable for cell therapy while reducing the amount of non-defined components. Lessons from developmental biology have identified signalling molecules that can guide the differentiation process *in vitro*, but less attention has been paid to the extracellular matrix used. With the introduction of more biologically relevant and defined matrices, that better mimic specific cell niches, researchers now have powerful resources to fine-tune their *in vitro* differentiation systems, which may allow the manufacture of therapeutically relevant cell types. In this review article, we revisit the basics of the extracellular matrix, and explore the important role of the cell–matrix interaction. We focus on laminin proteins because they help to maintain pluripotency and drive cell fate specification.

This article is part of the theme issue ‘Designer human tissue: coming to a lab near you’.

## 1. Introduction

Following the derivation of human embryonic stem cells (hESCs) in 1998 [1], and with the development of human induced pluripotent stem cells (hiPSCs) in 2007 [2,3], stem cell research is rapidly progressing from pre-clinical studies to the clinical arena with the first human trials having already begun. Continued growth in the cell therapy industry and the commercialization of cell therapies has led to an increased awareness of the need for specialized and defined materials.

Current practices for the maintenance and expansion of undifferentiated human pluripotent stem cells (hPSCs) typically depend on the support of undefined culture systems, such as feeder cells (often mouse embryonic fibroblasts) or undefined basement membrane (BM) extracts such as Matrigel™ and Geltrex^®^ containing laminin-111, entactin, collagen, heparin sulfate proteoglycans and growth factors purified from Engelbreth–Holm–Swarm mouse sarcoma. Besides the risk of transmitting pathogens and the introduction of tumour-derived growth factors, culture conditions that depend on these undefined support systems limit experimental reproducibility and the ability to interpret mechanistic studies owing to lot-to-lot variations. Ultimately, they hinder the transition of hPSC-derived products into a clinical setting.

Over the past decade, several feeder-free, extracellular matrix (ECM) protein culture matrices have appeared on the market that are defined and xeno-free [4–6]. However, not all ECM proteins are suitable for hPSC culture because they cannot maintain an undifferentiated hPSC population [7] or allow clonal survival of hPSCs without the use of apoptosis inhibitors or undefined additives [8]. Moreover, hPSCs were often passaged as clumps on these substrates, which significantly induced the risk of spontaneous differentiation [8]. High production costs, labour-intensive cell passaging and the limited scale-up potential associated with many of these ECM substrates have driven researchers to seek alternative substrates in the synthetic polymer arena. For example, Synthemax™, an acrylate surface with deposits of various peptide–polymer conjugates, has been developed for the adhesion and expansion of hESC [9]. Synthetic scaffolds composed of poly(desaminotyrosyl tyrosine ethyl ester carbonate) [10], a tyrosine-derived polycarbonate polymer [11], have also been used to study the effects of geometry on hESC survival and self-renewal. Carlson *et al.* have shown that poly-d-lysine pre-treated scaffolds support hESC survival and colony formation. However, the authors also illustrated the importance of cell–ECM interactions for cell functionality and concluded that the endogenous cell production of laminin was an essential factor for adhesion and survival of the hESCs [10].

A lack of biologically relevant signals from the matrix increases the risk for mixed cell populations and genetic and phenotypic drift *in vitro*. Compared with many other feeder-free matrices on the market, full-length laminin-521 better mimics the natural environment for hPSCs in culture [7,8,12]. Owing to their biologically relevant interactions with cells, laminin-based substrates have significantly advanced the stem cell research field and are, therefore, the focus of this article. Over the past decade, laminins have become a preferred cell culture substrate both within basic research as well as for researches and companies with a therapeutic focus [13].

## 2. The ECM and the importance of biological relevance for cell culture

**Figure 1.**
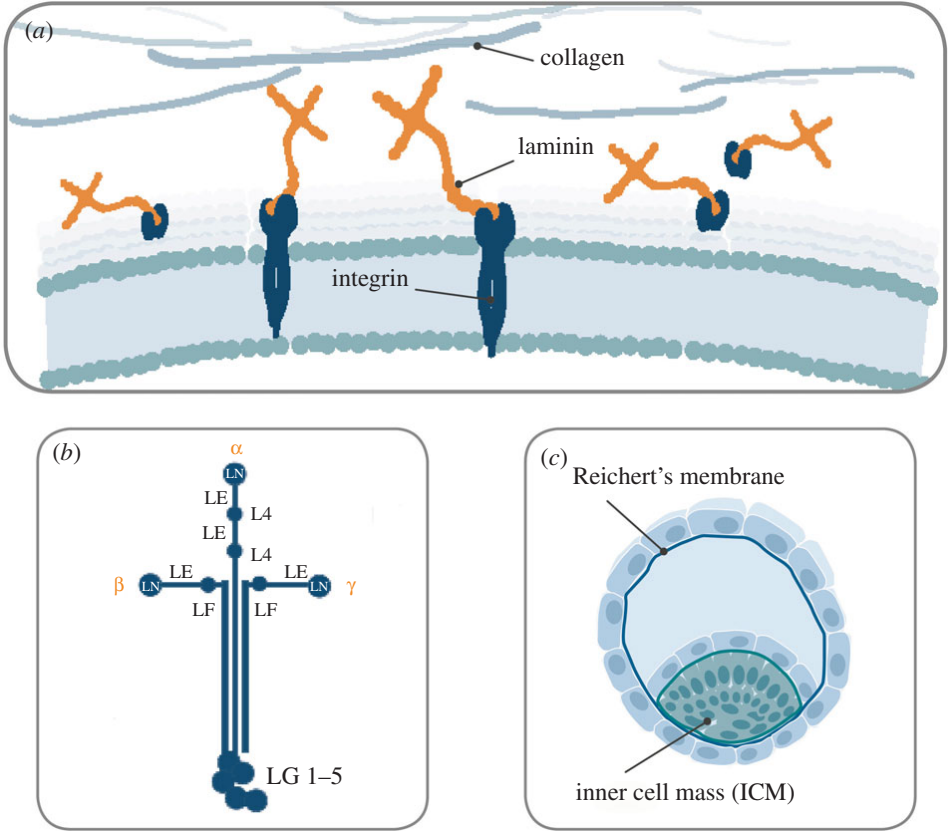
(*a*–*c*) Laminins are abundant components of the BMs and have essential roles in structural organization. Via binding to cell-surface receptors, such as integrins, laminins are also key regulators of cell behaviour (*a*). The molecular structure of laminin isoforms is cross-shaped. The Greek letters indicate the specific chains. The α chains have five globular domains (LG1–5) at the C terminus. The short arms are composed of a globular laminin N-terminal domain (LN), globular laminin IV domain (L4), or laminin four domain (LF). Each of these domains is separated by rod-like regions formed by multiple laminin epidermal-growth-factor-like (LE) domains (*b*). The α5-chain laminins are key cell adhesion proteins of the natural stem cell niche. It is naturally expressed and secreted by cells in the inner cell mass ICM of the embryo whereas the α1-chain laminins are mostly expressed in the Reichert's membrane which supports the outer extra-embryonic layer of trophoblasts (*c*).

Most organized and stationary cells of the body grow on specialized, sheet-like ECM structures called basement membranes (BMs). Different cells and tissues require a specific ECM composition for survival and proper function [14], and many cells, such as fibroblasts [15], endothelial cells [16] and cardiomyocytes [17], themselves produce and deposit ECM proteins. The BM contains specific, highly conserved proteins, and consists mostly of laminin, type IV collagen, agrin, perlecan, fibronectin and nidogen [14,18] (figure 1*a*).

Over the past 40 years, several adhesion molecules (fibronectin, vitronectin, laminin-111 or a placenta-derived mixture of laminins) have been discovered and used to improve cell cultures [19,20]. The concept of adhesive matrix molecules has gradually improved: adhesion is not only for anchoring cells in place but is also crucial for cell survival and phenotype maintenance. Cells that lack their relevant ECM cues undergo anoikis, controlled suicide, which is a natural mechanism that allows for the correct organization of tissues and for the selection of specific cell types. Laminin and type IV collagen form networks that have a structural role, influence cell migration and regulate the differentiation processes of associated cells. Nidogens are sulfated glycoproteins that link the BM proteins together, and the glycosaminoglycan-containing perlecan and agrin bind growth factors and contribute to the matrix volume [12]. Laminins are the most abundant component of the BM. In addition to their structural functions, laminins play an essential role in the structural organization of the BM and the regulation of cell behaviour [21].

Laminins are multidomain, heterotrimeric glycoproteins, composed of three different subunits: an α-chain, β-chain and γ-chain, combined and expressed in at least 16 different isoforms in the human body [12,18]. The physical, topological and biochemical expression of the different laminin isoforms in the BM is heterogeneous and laminin expression changes during development. Laminin-111 is widely expressed during embryogenesis, but its tissue distribution after birth is restricted to only a few tissues, such as the brain and kidney [22]. After birth, the α5-laminins are most common [23]. Without the right combination of laminin isoforms, cells and tissues become dysfunctional. While epithelial cells need laminin-332 together with laminin-511/521 for proper function, muscle and nerve cells require laminin-211, -221 and laminin-511/521, and endothelial cells prefer a combination of laminin-411/421 and laminin-511/521 [12].

The molecular structure of laminins is cross-shaped with the N-terminal ends forming three short arms, containing globular laminin N-terminal domains and a globular laminin IV domain [24,25]. Each of these domains is separated by rod-like, epidermal-growth-factor-like domains, harbouring nidogen binding sites [26]. Nidogen and perlecan in turn, allow the laminin network to be connected to collagen IV. The globular N-terminal domains also bind to syndecans which regulate cell adhesion and motility [27,28] (figure 1*b*). Cells bind to laminins via cell-surface receptors, such as integrins, which regulate vital cellular responses, such as proliferation and differentiation, migration, phenotype stability and resistance to apoptosis [12,29,30]. The α5-laminins (especially laminin-511 and laminin-521) exhibit the broadest degree of integrin binding interactions [23]. There are many binding sites on the full-length laminin molecule that can interact with cell membrane receptors; however, the molecular mechanism underlying the laminin-mediated signalling to integrins and other cell-surface receptors is still not fully elucidated. Studies indicate that the LG domains in the C-terminal region of laminins are highly involved in laminin recognition by integrins [31,32]. Integrins, α-dystroglycan, perlecan and sulfated glycolipids also interact with some of the globular N-terminal laminin domains [18,33]. Fractionated laminin molecules isolated from tissue and truncated laminin molecule derivatives lack most of the laminin domains that are required for the formation of a proper extracellular network. Moreover, full-length recombinant laminins with all their native biological activities intact are essential for biomimetic *in vitro* function [34] and for the stimulation of authentic cellular signal transductions. Emphasizing biology and mimicking the natural matrix proteins is one of the most important aspects to create a biologically relevant milieu for the cells, resulting in phenotypically stable cell cultures and reproducible protocols.

## 3. Biologically relevant cell culture matrices enable clinical translation of research protocols

Advancing a PSC-derived cell therapy from pre-clinical studies to a phase 1 clinical trial requires a demonstration of a well-controlled production process and a safe and efficacious product to the regulatory agencies. The development of a differentiation protocol that generates the target cell type at a sufficient quantity and purity, with phenotypic maturity and appropriate cellular functions, is arguably challenging. Owing to their validated functionality and biological properties, human recombinant laminins in conjunction with streamlined differentiation protocols offer exciting prospects for regenerative medicine. This has been highlighted in a number of high-impact scientific articles in the past two years and a few examples are described below.

### (a) PSC derivation, maintenance and safety

In the developing embryo, laminins containing the α1- and α5-chain are the first ECM proteins to be expressed. They are essential for early embryogenesis and initiation of morphogenesis [21]. α5-Chain laminins (i.e. laminin-521 and laminin-511) are produced by and surround the cells in the inner cell mass of the blastocyst which gives rise to all embryonic tissues [12,14,35] (figure 1*c*). α5 Laminin is also produced endogenously by pluripotent hPSCs cultured *in vitro* and is a critical autocrine and paracrine factor that regulates hPSC survival and self-renewal. Knockdown and disruption of the *LAMA5* gene dramatically reduces hPSC self-renewal and increases apoptosis [7]. LN-521 thus constitutes the relevant niche for pluripotent stem cells when cultured *in vitro.* Laminin-111 is mostly expressed in the Reichert's membrane, which supports the outer extra-embryonic layer of trophoblasts and is widely expressed during embryogenesis [14,36] (figure 1*d*). Hence, using cell culture substrates that contain laminin-111 (e.g. Matrigel or Cultrex) is suboptimal for reliable survival and expansion of hESCs and hiPSCs [8,12,29]. Human ESCs dissociated into single-cell suspension and plated on Matrigel, or different human recombinant laminin (LN) substrates, LN-111, LN-121, LN-511 and LN-521, in the presence of apoptosis inhibitor (ROCK inhibitor, Y-27632), survive on all surfaces. However, in the absence of ROCK inhibitor, hESCs do not survive well on Matrigel, LN-111 or LN-121, whereas they readily adhere and spread on LN-511 and LN-521 [8]. By culturing cells on the laminin isoforms that they normally adhere to *in vivo in vitro*, the biologically relevant niche is better recapitulated by the medium and the matrix.

**Figure 2.**
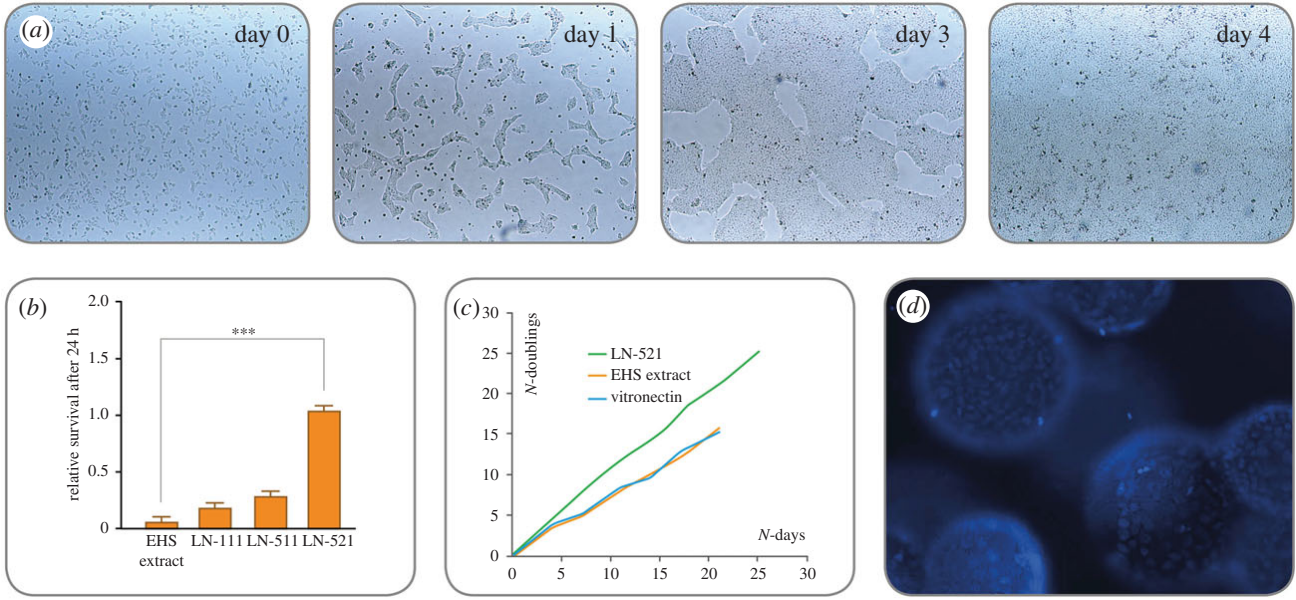
(*a*–*d*) hPSCs grow as homogeneous monolayers on LN-521, exhibiting a high nuclear-to-cytoplasm ratio and prominent nucleoli. Owing to the biologically relevant support from the matrix, hESCs and hiPSCs can be seeded as single cells on LN-521 without the need to use apoptosis inhibitors (e.g. ROCKi) for survival. Within 1 h after seeding, the cells have attached and are evenly distributed across the surface (*a*, day 0). The cells show high motility on LN-521, migrate and form small colonies and start dividing (*a*, day 1). The colonies merge (*a*, day 3) into a homogeneous monolayer without signs of spontaneous differentiation (*a*, day 4). LN-521 supports higher post-seeding survival (24 h) of single-cell dissociated hPSCs, compared with Matrigel and non-relevant laminin matrices (*b*). In addition, hPSCs proliferate faster on LN-521 compared with other feeder-free matrices (EHS extract; Engelbreth–Holm–Swarm mouse sarcoma extract), with about 10-fold increase every 4 days (*c*). LN-521 even enables large-scale automated processes for robust hPSC expansion in bioreactors. The cells easily attach, spread evenly on the surface of the microcarriers and give a 20-fold expansion within 4 days (*d*).

In 2014, Rodin *et al.* [37] first described an efficient xeno-free and chemically defined protocol for monolayer culturing of hPSC on LN-521 (figure 2*a*). Via the interaction to specific hPSC cellular receptors, LN-521 triggers authentic cell signalling pathways, which promotes high survival (figure 2*b*) and robust long-term expansion of single-cell cultured hESCs and hiPSCs (figure 2*c*), even without the addition of ROCK inhibitor or any other inhibitors of anoikis. LN-521 binds to β1 integrin receptors, primarily α6β1, which plays a pivotal role in the stem-cell–matrix interaction. hPSCs predominantly express the α6β1 integrin, which interacts strongly with LN-521 globular domains inducing the PI3 K/Akt pathway [8,38,39]. The inactivation of the focal adhesion kinase (FAK) signalling pathway via the α6β1 integrin is also linked to the hPSC self-renewal and expression of pluripotency [40]. The motility of hPSCs on LN-521 is higher than that of the cells on other matrices, which correlates to the high survival of the cells [8].

hPSCs in single-cell suspension plated on LN-521 quickly attach, migrate and form monolayer colonies which rapidly expand (figure 2*a*). hPSCs cultured in thick multilayer colonies often require manual, arbitrary removal of spontaneously differentiated parts. By contrast, hPSCs cultured on LN-521 grow as a homogeneous monolayer, with minimal risk of spontaneous differentiation or genetic abnormalities [41]. The biologically relevant culture environment generates hPSC cultures with a more uniform gene expression between different hPSC lines [42] and enhances cell maturation, polarization and functional organization in many cell applications [43–45].

The LN-521 matrix also allows efficient clonal derivation, clonal survival and long-term self-renewal of hESCs. Human ESC lines can even be derived from a single blastomere under chemically defined and xeno-free conditions [8,13], which circumvents the ethical issues associated with embryo destruction for hESC line derivation. The biologically relevant culture environment provided by the LN-521 substrate allows for flexible culture protocols that support weekend-free feeding [41], ultra-low seeding density (greater than 5000 cells cm^−2^) and high confluence culture.

### (b) Stem cell-derived dopamine progenitors from a high-yield good manufacturing practice-adapted differentiation protocol

Parkinson's disease is an interesting target for cell replacement therapies owing to its focal degeneration of midbrain dopamine (DA) neurons. Proof-of-concept has been achieved in clinical trials using human foetal ventral mesencephalic (VM) tissue [46]. However, the low availability of such tissue limits its use, and a renewable source of cells is required in order to develop a therapy that is accessible to a large number of patients.

A new differentiation protocol that efficiently and rapidly patterns hESCs to midbrain DA neurons using extrinsic patterning agents has been developed in recent years [47,48]. These stem cell-derived DA neurons function *en par* with foetal midbrain DA neurons [49]. Kirkeby *et al.* have developed a fully defined and xeno-free protocol. Even so, a number of steps were required to develop a good manufacturing practice (GMP) version compliant with use in clinical trials [45,50]. One important step was to switch from an initial suspension culture step to a fully attached protocol. Matrigel had previously been used for this purpose [33] but is not ideal for GMP manufacturing; more suitable substrates, such as recombinant laminins, were required for GMP production.

Seven different recombinant laminin isoforms were screened for their ability to replace Matrigel/free floating suspension cultures and four of them were found to efficiently support adherent differentiation of VM progenitors (LN-111, LN-421, LN-511 and LN-521). It has previously been reported that LN-511 and LN-521 efficiently support growth of hPSCs [8], making them less ideal in this differentiation protocol. In contrast, undifferentiated hESCs detach from LN-111-coated culture dishes when kept in pluripotency medium but efficiently attach in neural differentiation medium making it an ideal substrate to move forward with.

**Figure 3.**
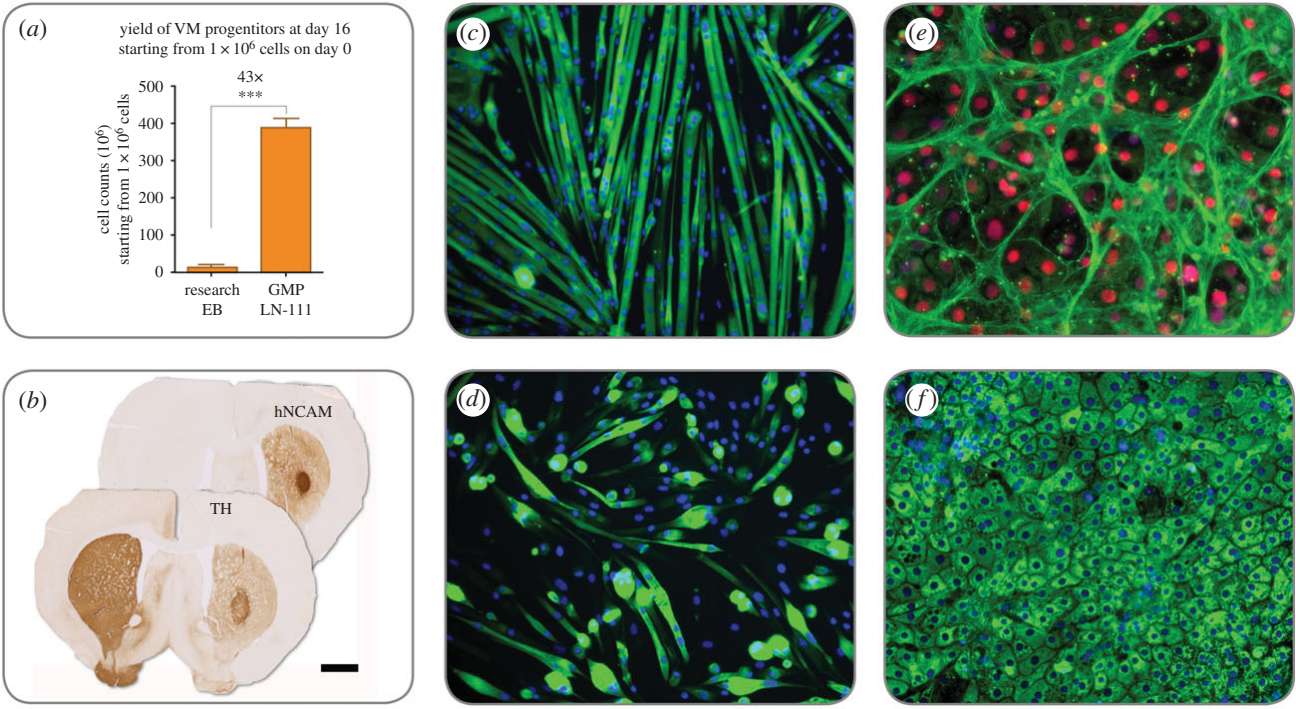
(*a*–*f*) Differentiation of hESCs on LN-111 towards DA cells results in more than a 40-fold increase of transplantable DA progenitors at day 16 (*a*) and the human progenitors innervate the striatum, become tyrosine hydroxylase (TH+) neurons and contribute to complete behavioural recovery after transplantation into animals modelling Parkinson's disease (*b*; scale bar represents 1.5 mm). LN-521 improves muscle cell proliferation and differentiation, giving larger myotubes, higher amounts of nuclei per myotube (express myosin heavy chain, MHC) and more consistent differentiation in long-term cultures (*c*; MHC in green) compared with cells on Matrigel (*d*; DAPI in blue). Human ESCs differentiated on LN-521 and LN-111 demonstrate efficient hepatocyte maturation with significant improvements in cell function [43]. The hepatocyte-like cells are highly organized on LN-521, express transporter protein MRP1 (*e*; green) and HNF4a (red), and a majority of the cells are albumin+ (*f*; green) Cell nuclei are visualized with DAPI (blue).

When implementing this in the GMP protocol, the differentiation on LN-111 resulted in robust and reproducible differentiation of midbrain DA progenitors with minimal variation between batches [45,50]. Moreover, the yield was greater than 40 times the original research grade differentiation protocol [45,48] (figure 3*a*). This high yield means that it is possible to produce greater than 3.8 × 10^8^ transplantable progenitor cells in 16 days when starting from just 1 × 10^6^ undifferentiated hESCs [45] (figure 3*b*). This would provide more than 500 doses of cryopreserved DA neurons from one manual manufacturing batch at a relatively small scale.

### (c) Recombinant laminins support the differentiation potential of satellite cell-derived myoblasts during long-term culture

Every tissue in the body originates from a stem cell during development but there are a number of tissues where stem cells exist to facilitate tissue regeneration in the adult. In skeletal muscle, that stem cell is called the satellite cell and it remains quiescent unless stimulated by muscle damage [51]. Upon damage, it enters a programme to asymmetrically expand to generate a transit amplifying cell and another satellite cell [52,53]. The transit amplifying cell undergoes several rounds of division to create a population of cells called myoblasts that can fuse together to create new muscle fibres or fuse to existing fibres to repair membrane lesions.

Large-scale expansion of muscle stem cells *in vitro* is critical for a variety of purposes. Large quantities of satellite cells are required for cell engineering, cell therapy and to support skeletal muscle drug discovery campaigns. Relatively small numbers of primary satellite cells must be scalable to millions, even billions, of cells while maintaining the ability to differentiate into mature myotubes. While multiple substrates have been commonly used for growth of satellite cells, long-term effects of culturing on different substrates have not been well characterized. Matrigel is commonly used; however, it is a complex substrate exhibiting significant lot-to-lot variation, and variable amounts of growth factors that may skew experimental outcomes and mitigate reliable translation of protocols from the literature to drug discovery applications [54]. Additionally, Matrigel implementation varies largely from laboratory to laboratory, making it difficult to reliably translate protocols from the literature for drug discovery applications. In order to develop an optimized and defined culture platform, an in-depth comparison of long-term satellite cell activity between multiple defined extracellular matrices and Matrigel has been performed. It was shown that LN-521 dramatically enhances the proliferation and differentiation of satellite cells and extends their culture potential well past previously established passaging limits [44]. Following eight passages *in vitro*, LN-521 expanded satellite cells form more mature myotubes containing high numbers of nuclei and better organization compared with the other substrates (figure 3*c*). In contrast, both LN-111 and Matrigel expanded cultures contain smaller immature myotubes with small numbers of nuclei (figure 3*d*), an effect observed with both human and mouse-derived satellite cells. Overall, it was demonstrated that LN-521 provides a good substrate that enables large-scale expansion in a shorter time while maintaining a differentiation performance that better resembles freshly isolated cells [44].

Additional work from Penton and colleagues has demonstrated that cells grown on LN-521 show enhanced engraftment into skeletal muscle niches *in vivo* (unpublished data). Although there have been many publications that suggest the limited utility of satellite cells for cell therapy, those experiments may have been flawed because they employed satellite cells that had already lost some of their expansion and differentiation potential *in vitro*. Interestingly, satellite cell markers including pax7 and integrin alpha 7 were expressed at similar levels across substrates, suggesting that LN-521 is regulating satellite cell activity through novel mechanisms (C. Penton and P. August, unpublished data). Plating satellite cells on LN-521 immediately after isolation by fluorescence-activated cell sorting (FACS) appears to fundamentally protect the cells from the loss of the expansion and differentiation abilities. Penton and colleagues are investigating additional approaches that should permit enhanced expansion and engraftment to develop advanced therapeutic approaches for patients with muscular dystrophies.

Previous studies have shown that transplanted hPSC-derived myogenic cells can fuse with host myofibres and improve muscle function [55]. However, it is unknown if hPSC-derived myotubes alone can generate three-dimensional functional skeletal muscle because comparison studies suggest they are more developmentally immature than primary myotubes. By trying to mimic the tissue architecture both with biologically relevant matrix molecules and different three-dimensional approaches, it is possible to generate functional biomimetic skeletal muscle tissues entirely from hPSC-derived myogenic cells [56].

### (d) Recombinant laminins drive the differentiation and self-organization of hESC-derived hepatocytes

There has been tremendous progress over the past 15 years in generating pure populations of endodermal cells, including hepatocytes, for biomedical application [57]. While cell-based models improve the ability to model human liver biology ‘in the dish’ and may be suitable for cell-based therapy, most approaches rely on undefined materials in the differentiation process [58]. This severely limits technology reproducibility and scale up, acting as a barrier to technology translation.

In recent years, serum-free and chemically defined differentiation procedures have been developed [59] that have been validated by the pharmaceutical industry to be as good as their current gold standard [60]. While this marked progress at the time, the early prototype systems still relied on poorly defined ECMs, such as Matrigel, which resulted in batch-to-batch variation. Since these studies, a differentiation system has been designed using polymer library screening to identify new polyurethane substrates for cell culture [61,62].

More recently, differentiation has been performed using recombinant laminins [43]. This enabled a highly reproducible and transformative differentiation process, which has been semi-automated to permit large-scale manufacture. Importantly, cells generated in this manner express proteins, HNF4a, MRP1 and albumin, found in hepatocyte (figure 3*e,f*). An essential part of the stem cell scale-up is the ability to use highly defined ‘off the shelf reagents'.

## 4. From here to the future: enabling cell therapy

Pluripotent stem cells, whether they are derived from human embryos or reprogrammed adult or foetal somatic cells, have an intrinsic capability for unlimited self-renewal and the ability to make all the cells in the body. They are, therefore, an ideal candidate to be used as starting material for cell therapies. Much effort has been put into creating methods for hPSC expansion that are defined, robust, simple and safe [63]. Having a defined method ensures low variance, high-quality, traceability and control over reagents, all required for the manufacture of clinical-grade cells. The robustness of the method allows consistency of results and predictability. A simple and controlled method reduces cost, minimizes the risk for mistakes and will more likely be adapted by users. Ultimately, the most important aspect of the hPSC culture method is to create a safe cell source. With extended time in culture, cells may begin to drift genetically, resulting in chromosomal abnormalities. Although hPSCs have an impressive ability to maintain genomic integrity over time, chromosomal abnormalities often occur at higher passages and/or as a result of suboptimal culture conditions [41,64,65].

### (a) Cell therapy-grade cell culture substrates

The stem cell therapy field is still in its infancy. Translational researchers are now looking more intensely at the quality and character of both the cell products and the culture products used for primary, stem and differentiated cells. So far, the US Food and Drug Administration and the European Medicines Agency have not demanded GMP for phase 1 studies but it is evident that the methods employed today will not be sustainable for commercial therapies. As a consequence, the conversion of optimized, scalable and standardized culture methods into GMP-compatible protocols will become another challenge. Each and every reagent needs to be high quality, traceable, controlled and preferably manufactured according to GMP. To address these issues, a cell therapy-grade (CTG) LN-521 substrate has been developed to enable cell therapy fast-forward to an affordable price. The CTG LN-521 substrate is stably expressed and produced by CAP^®^ Go cell, a suspension-based platform already used for therapeutic proteins in human clinical trials, and is a validated source of protein of ethically accepted origin. A qualified GMP master cell bank together with a scalable and regulatory compliant production process of CTG LN-521 serve as the foundation for translational research and cell manufacture.

### (b) Cell culture at the appropriate scale and with automation

Generation of hESC and hiPSC lines is laborious. To decrease the amount of work needed, increase success rates and minimize hazardous deviations, the process needs to be standardized. In addition, an automated process is desirable to avoid subjective, operator-dependent mistakes and to cut costs.

Generally, upscaling is achieved by manual expansion in traditional culture vessels or through scalable planar flask cultures, which all involve multi-layered stacked-plate systems. Automation of traditional culture vessels, such as plates and flasks, can be achieved by pipetting (semi- or whole) closed system robotics. Another approach is packed-bed bioreactors which are perfusion-based three-dimensional scaffolds. A further type of bioreactor-supported system offering the precise control of the culture environment is microcarrier culture in stirred tanks, ideal systems for achieving required lot sizes. The surface-area-to-volume is greatly increased and thus renders more cells with less medium, reagents and materials [66].

Stem cell scale-up and subsequent differentiation would ideally be performed in the same, closed, automated and fully controlled bioreactor. Human recombinant laminin can easily be adsorbed by microcarriers (e.g. Corning^®^ Enhanced Attachment Microcarriers) and LN-521 coated microcarriers have been shown to sustain expansion of both hESC and hiPSCs in stirred tank bioreactors, with sequential differentiation to cardiomyocytes [67] (figure 2*d*).

### (c) Importance of biological relevance: spatial, temporal and regional impact

The development of robust protocols that safely and accurately direct hESCs and hiPSCs into the desired cell types, mimicking the signalling that occurs during development is difficult to achieve *in vitro*. While many protocols successfully recapitulate the temporal developmental processes via well- designed medium formulations, the majority of protocols ignore the role of the ECM and the spatial and tissue-specific distribution of the ECM proteins that form the tissue architecture.

One of the biggest and most obvious differences between cells cultured in two-dimensions versus three-dimensions is their morphology. The orientation of the integrin-mediated adhesions guides the orientation and thus the shape of the cells. A three-dimensional microenvironment influences the attachment, spreading, growth and most importantly cell polarity [68,69]. The limitations of two-dimensional cultures have contributed to poor predictive power of pre-clinical cell-based drug and toxicity screening assays. Human recombinant laminins, in combination with other key ECM molecules, can be used together with various available three-dimensional solutions. Hydrogels can be covalently linked with proteins and thus serve as a three-dimensional context for the cells. In addition, recombinant silk proteins can be used as scaffolds that can easily be adsorbed with, for example, LN-521 to maintain and differentiate hESCs towards different lineages. In order to mirror the correct *in vivo* tissue assembly *ex vivo*, and to regenerate damage tissue and even repair whole body parts, biologically relevant and physiological matrices in a structure allowing spatial orientation need to be developed.

This article has been designed to create an awareness around the necessity and need for defined materials to improve basic scientific research and to permit the translation of these advances through reliable manufacture of cell-based products at a large scale. Additionally, we emphasize the importance of a close collaboration between the users, suppliers and regulators to facilitate the commercial development of safe and effective cell therapies globally.

## Data accessibility

This article has no additional data.
